# Effect of Human Corneal Mesenchymal Stromal Cell-derived Exosomes on Corneal Epithelial Wound Healing

**DOI:** 10.1167/iovs.18-24803

**Published:** 2018-10

**Authors:** Ravand Samaeekia, Behnam Rabiee, Ilham Putra, Xiang Shen, Young Jae Park, Peiman Hematti, Medi Eslani, Ali R. Djalilian

**Affiliations:** 1 Department of Ophthalmology and Visual Sciences, University of Illinois at Chicago, Chicago, Illinois, United States; 2 Department of Medicine and University of Wisconsin Carbone Cancer Center, University of Wisconsin-Madison, School of Medicine and Public Health, Madison, Wisconsin, United States

**Keywords:** exosome, corneal wound healing, mesenchymal stromal cells, corneal epithelium, wound healing

## Abstract

**Purpose:**

Mesenchymal stromal cells (MSCs) have been used therapeutically to modulate inflammation and promote repair. Extracellular vesicles, including exosomes, have been identified as one of the important mediators. This study investigated the effect of human corneal MSC-derived exosomes on corneal epithelial wound healing.

**Methods:**

Corneal MSCs (cMSCs) were isolated from human cadaver corneas. The secretome was collected after 72 hours and exosomes were isolated using differential ultracentrifugation. Morphology and size of exosomes were examined by electron microscopy and dynamic light scattering. Expression of CD9, CD63, and CD81 by cMSC exosomes was evaluated by western blotting. Cellular uptake of exosomes was studied using calcein-stained exosomes. The effect of exosome on wound healing was measured in vitro using a scratch assay and in vivo after 2-mm epithelial debridement wounds in mice.

**Results:**

cMSC exosomes were morphologically round and main population ranged between 40 and 100 nm in diameter. They expressed CD9, CD63, and CD81, and did not express GM130, Calnexin, and Cytochrome-C. Stained cMSC exosomes were successfully taken up by human cMSCs, human corneal epithelial cells (HCECs), and human macrophages in vitro and by corneal epithelium in vivo. In scratch assay, after 16 hours, cMSC exosome treated HCECs had 30.1% ± 14% remaining wound area compared to 72.9% ± 8% in control (*P* < 0.005). In vivo, after 72 hours, cMSC exosome-treated corneas had 77.5% ± 3% corneal wound healing compared to 41.6% ± 7% in the control group (*P* < 0.05).

**Conclusions:**

Human cMSC exosomes can accelerate corneal epithelial wound healing, and thus, may provide a therapeutic approach for ocular surface injuries.

Nonhealing corneal wounds are an important cause of loss of vision in the setting of ocular surface diseases.^1^ While there has been great progress in the treatment of corneal diseases, wound healing in the setting of severe corneal disease or damage still remains challenging.^2^ Conventional management of corneal wounds consists mainly of supportive measures in the form of lubrication, antibiotics, and bandage contact lenses followed by amniotic membrane and tarsorrhaphy for recalcitrant cases. Recent studies have shown the beneficial effects of stem cell-based therapies on ocular surface healing, particularly using mesenchymal stem/stromal cells (MSCs).^3–6^

MSCs are found in most adult tissues, including cornea/limbus,^7^ and play an important role in tissue repair and maintenance.^1^ Over the past decade, they have been increasingly investigated for their therapeutic potential in a wide range of human diseases given their anti-inflammatory and regenerative properties. It is now widely accepted that the therapeutic effects of MSCs are mediated largely through their secreted factors.^7–11^ We previously showed that human corneal MSC (cMSC) secretome is able to inhibit corneal neovascularization,^7^ and modulate macrophages toward an anti-angiogenic and anti-inflammatory immunophenotype,^12^ suggesting that human cMSC secreted factors may be used therapeutically in ocular surface diseases. Secreted factors present considerable advantages over cells regarding manufacturing, storage, handling, product shelf life and their potential as a ready-to-go biologic product.^13^ Therefore, recently the use of secreted factors derived from MSCs, instead of using the cells, has attracted attention for their potential advantages in tissue repair and regeneration.^10,11,13–16^

Previous studies have identified exosomes as one of the important secreted constituents in mediating and carrying out the biological functions of MSCs.^17–19^ Exosomes, bi-lipid membrane bound nanovesicles with a size range of 40 to 100 nm, are known to transfer bioactive molecules among cells and play crucial roles in wound healing and angiogenesis.^15,20,21^ In the present study, we isolated human cMSC-derived exosomes to investigate their role, as a potential therapeutic for promoting corneal epithelial wounds.

## Methods

### Isolation and Culture of Human cMSCs

Human cMSCs were cultured from human cadaver corneas as described before.^7^ Corneoscleral buttons from healthy cadaver eyes were kindly provided by Eversight. After removing the central cornea with an 8.5-mm trephine, the limbal rims were washed five times in PBS and placed in 2.4 IU of Dispase II (Thermo Fisher, Waltham, MA, USA) for 1 hour at 37°C. The next day, after removing the epithelium from the limbal rim, it was cut into small pieces and incubated in collagenase Type I (0.5 mg/mL) (Sigma-Aldrich Corp., St. Louis, MO, USA) in Dulbecco's modified Eagle's medium/F12 media (Thermo Fisher) overnight at 37°C. The digests were filtered through a 70-μm nylon strainer and the single-cell suspension was seeded onto a six-well tissue culture plate in α MEM media supplemented with 10% fetal bovine serum, 1X L-Glutamine, and 1X NEAA (all from Thermo Fisher). The media were changed every other day, and cells were subcultured by brief digestion with TrypLE Express (Thermo Fisher) when 80% confluent. As reported by our group previously, cMSCs isolated in this fashion express MSC markers defined by International Society of Cell Therapy and demonstrate trilineage differentiation.^7^

### Isolation of Exosomes

Exosomes were isolated using ultracentrifugation method. Briefly, human cMSCs were cultured in α MEM media supplemented with 10% fetal bovine serum, 1X L-Glutamine, and 1X NEAA (all from Thermo Fisher) until confluence. The confluent cells were washed three times with PBS, and then the media was changed to serum free, phenol red-free α MEM (Thermo Fisher). After 72 hours, the conditioned medium (CM) from the cultures was collected and to eliminate cell debris and macroparticles, was centrifuged at 400*g* for 10 minutes, then the supernatant was collected and centrifuged at 1500*g* for an additional 30 minutes. The supernatant was collected and concentrated by ultra-filtration with a 100-kDa molecular weight cut-off membrane (Thermo Fisher). The concentrated CM was ultracentrifuged at 100,000*g* for 2 hours. Pellet was resuspended in PBS and was ultracentrifuged at 100,000*g* for 2 hours. The final pellet was resuspended in PBS with a volume ratio of exosome to CM being 1:1000 and used right away or stored in 4°C until further use for no more than 72 hours.

### Analysis of Exosome Morphology, Size Distribution, and Quantification

#### Transmission Electron Microscopy

A 15-μL droplet of exosome solution was adsorbed to Formvar/carbon coated copper grids. The exosomes were then negatively stained using 2% aqueous phosphotungstic acid (PTA), blotted, and air dried. A JEOL JEM-1220 TEM with an accelerating voltage of 80 kV36 was used for examination and imaging.

#### Dynamic Light Scattering (DLS)

The size and polydispersity of the isolated exosomes were determined using NanoSight (Malvern zetasizer, Worcestershire, UK). One hundred microliter suspensions of exosomes were diluted to 1000 μL in PBS, added to a cuvette, and the air bubbles were carefully removed. Three scattering measurements for size and density were recorded.

#### Exosome Quantification Assay

Exosomal concentration was assessed using the EXOCET assay (System Biosciences, Mountain View, CA, USA), according to the manufacturer's instructions. This is an enzymatic colorimetric assay measuring the absorbance at 405 nm of esterase activity known to be within exosomes. The assay was calibrated using a standard exosome preparation (System Biosciences).

### Western Blotting

Exosome pellets were resuspended in lysis buffer, and cMSC whole cell lysate was used as control. The lysates were boiled in sodium dodecyl sulfate (SDS) loading dye for 10 minutes and subjected to 4–20% SDS-polyacrylamide gel electrophoresis (PAGE). After blocking with 5% dry milk for 1 hour, the membranes were probed overnight with antibodies against CD9, CD63, CD81, and HSP70 (Cat# EXOAB series, System Biosciences) to represent positive markers for exosomes,^22^ and GM130 (Cat#2296, Cell Signaling, Danvers, MA, USA), Calnexin (SPA-860, StressGen, San Diego, CA, USA), and Cytochrome-C (Cat# 556433, BD Biosciences, San Jose, CA, USA) to represent negative markers for exosomes.^22^ After washing in 1% Tris-buffered saline-Tween 20 (TBST), membranes were incubated for 1 hour in appropriate peroxidase-conjugated secondary antibodies (1/5000 in all cases) and immunoreactivity was visualized with an electrochemiluminescent detection kit (Amersham, Buckinghamshire, UK) on a Li-Cor Odyssey system (Li-Cor, Lincoln, NE, USA).

### Exosome Fusion Assay

To track the cMSC exosomes, they were stained with a green fluorescent dye (Calcein-AM, Thermo Fisher). Briefly, 100 μL exosome suspension containing 1.0 × 10^8^ exosomes were mixed with 1 × 10^−6^ M dye and incubated for 30 minutes at 37°C. They were then resuspended in 8 mL PBS and ultracentrifuged at 100,000*g* for 1 hour. The pellets were resuspended in 100 μL PBS and used right away or stored in 4°C until further use for no longer than 72 hours. For human corneal epithelial cell (HCEC) studies, we used the telomerase immortalized human corneal limbal epithelial (HCLE) cell line (kindly provided by Ilene Gipson, PhD). The cells were grown in keratinocyte serum free media (KSFM; Thermo Fisher), seeded onto four-well culture slides and incubated at 37°C until they reached a confluence of 60% then exposed to the calcein stained exosomes for 4 hours. Similarly, human macrophages cultured from human buffy coats^12^ were exposed to stained exosomes for 4 hours, followed by fixation and imaging.

### In Vitro Scratch Wound Assay

HCECs were grown to confluence on 12-well plates in KSFM media (Thermo Fisher). The cells were growth factor–starved overnight and the cell monolayers were scratched using a sterile 200-μL pipette tip, and washed twice with PBS to remove the floating cells. They were then treated for 16 hours with either 1.0 × 10^8^ exosome per mL of media or the same amount of media mixed with PBS. The scratch area was captured serially using a spinning disc confocal microscope (Z1; Carl Zeiss Meditec, Jena, Germany), and photographed with an AxioCam camera (Carl Zeiss Meditec). The remaining wound area was measured using ImageJ software.^23^

### In Vitro Cell Proliferation Assay

HCECs were plated in a 96-well plate and grown to 60% confluence. They were growth factor starved and then treated with 1.0 × 10^5^, 1.0 × 10^6^, or 1.0 × 10^7^ exosome in each well (100 μL per well, triplicates per treatment) or vehicle control (PBS) for 16 hours. The media was then removed and 20 μL methylthiazol tetrazolium (MTT) was added into each well and incubated at 37°C for 3 hours; the solution was then removed and 50 μL dimethyl sulfoxide was replaced in each well. The concentration was determined by optical densitometry at the 560-nm wavelength.

### Corneal Debridement and Exosome Treatment

Corneal wounding experiments in mice were conducted in compliance with the ARVO Statement for the Use of Animals in Ophthalmic and Vision Research. The protocol was approved by the Committee on the Ethics of Animal Experiments of the University of Illinois at Chicago. Six-month-old C57BL/6J mice were anesthetized with intraperitoneal injection of ketamine (100 mg/kg) and xylazine (5 mg/kg). After applying topical 0.5% proparacaine, a 2-mm area of the central epithelium was demarcated and removed by an AlgerBrush II (The Alger Company, Lago Vista, TX, USA) as previously described.^23^ The wounded corneas were then treated topically with 5 μL of an exosome suspension containing 1.0 × 10^6^ exosome/μL, or the vehicle control (PBS) at 0, 10, 20, and 30 minutes time points. Wound closure was monitored at 0 and 24 hours using fluorescein staining and photographed using a camera-equipped Nikon FS-2 slit lamp biomicroscope. The percentages of wound closure were measured comparing with the baseline for each mouse, using ImageJ software.^23^ To examine the uptake of exosomes by the corneal epithelium in vivo, calcein-stained exosomes were applied topically in the same fashion onto scratch-wounded corneas of C57BL/6J mice. The corneas were then stained with 4′,6-diamidino-2-phenylindole (DAPI), mounted on a slide, and imaged with confocal microscope (Carl Zeiss Meditec).

### Statistical Analysis

All in vitro studies had at least three biological replicates. Results are presented as the mean ± standard deviation (SD) of three independent experiments. *P* values for differences were determined by double-sided nonparametric *t* tests, with GraphPad Prism software and Microsoft Excel. Differences were considered significant when *P* < 0.05. Error bars show SD of the mean.

## Results

### Characterization of Exosomes Isolated From Human cMSCs

**Figure 1:**
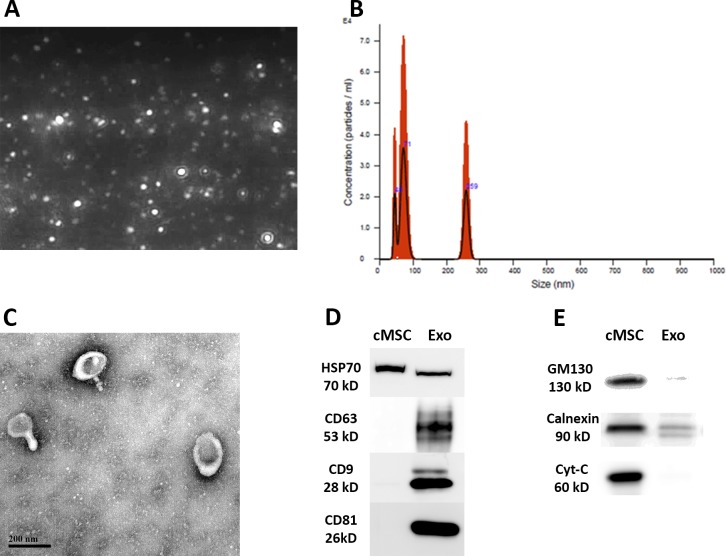
Characterization of exosomes. (A, B) DLS analysis, showing round particles with a size distribution of 40 to 280 nm. Most of the particles fall in the range defined for exosomes (40–100 nm). (C) Transmission electron microscopy of isolated exosomes showing a round morphology with a central depression, which is characteristic for exosomes. (D, E) Western blot results showing the presence of positive exosome markers CD9, CD63, CD81, and HSP70, as well as the absence or underrepresentation of negative exosome markers GM130, Calnexin, and Cytochrome-C, in isolated exosomes (Exo). cMSC whole cell lysate was used as control.

Exosomes from CM of human cMSCs were isolated by differential ultracentrifugation. The size distribution of exosomes was evaluated using DLS indicating a size distribution between 40 and 280 nm in diameter (Figs. 1A, 1B). More specifically, DLS demonstrated two populations, one more abundant population corresponding to exosomes (40–100 nm) and one less abundant population corresponding to larger size nanovesicles. TEM revealed exosomes with a round morphology and a characteristic central depression (Fig. 1C) and a size distribution consistent with exosomes. In fact, when we adjusted the results of size distribution with TEM, we observed that the vast majority of the particles were within the exosome size range (under 100 nm), in line with previous studies that revealed DLS falsely overestimates the amount of larger particles and the results should be confirmed with another method such as electron microscopy.^24–26^ Western blotting revealed the isolated exosomes to be enriched with endosomal tetraspanin proteins including CD9, CD63, and CD81 (positive markers for exosomes^22^) (Fig. 1D), while intracellular proteins including GM130, Calnexin, and Cytochrome-C (negative markers for exosomes^22^) were absent or highly underrepresented in the isolated exosomes (Fig. 1E).

### Human cMSC Derived Exosomes Are Taken Up by HCECs and Macrophages

**Figure 2:**
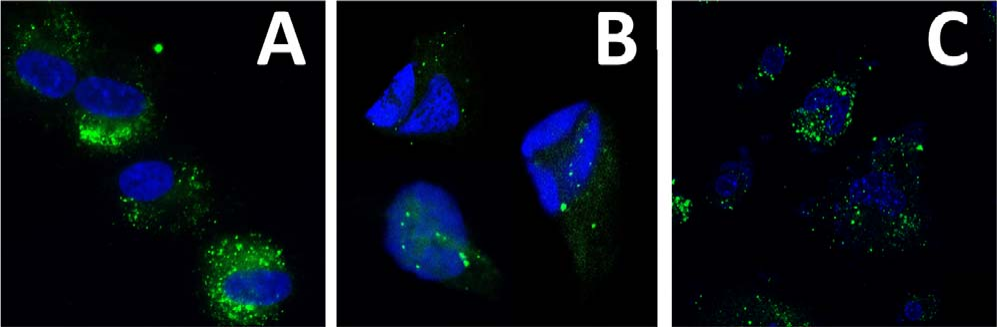
Uptake of cMSC derived exosomes by different type of cells in vitro. Cultured cells were incubated with calcein (green) labeled cMSC derived exosomes for 4 hours. Following incubation, fluorescent exosomes were taken up by human cMSCs (A), HCECs (B), and human macrophages (C).

We investigated the uptake of human cMSC exosomes by human cMSCs, HCECs, and human macrophages. After incubation of cultured cells with calcein labeled exosomes for 4 hours, green fluorescent particles were observed throughout the cells cytoplasm (Fig. 2). To determine the fate of the exosomes, the cells that had taken up the labeled exosomes were incubated for an additional 4 hours without exposure to exosomes. The results demonstrated a marked reduction in the number of fluorescent particles, suggesting that the exosomes had likely dissolved and released their content into the cells (data not shown).

**Figure 3:**
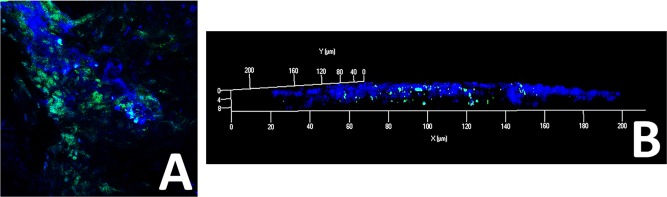
Uptake of cMSC derived exosomes by cornea epithelium in vivo. Mice corneas (after linear scratches) were topically treated with green labeled cMSC derived exosomes for 15 minutes under anesthesia. After 4 hours, imaging of the whole mount of the cornea showed wide distribution of exosomes throughout the mouse corneal epithelium (A). Z-stack imaging of the cornea whole mount showed penetration of the exosomes through the epithelium (B).

The uptake of exosomes was also examined in vivo following topical application to linearly scratched murine corneas. After 4 hours, whole mount imaging of the cornea showed wide distribution of labeled exosomes throughout the mouse cornea indicating successful fusion and uptake of cMSC exosomes by corneal epithelium in vivo (Fig. 3).

### Human cMSC Derived Exosomes Induce Wound Closure In Vitro

**Figure 4:**
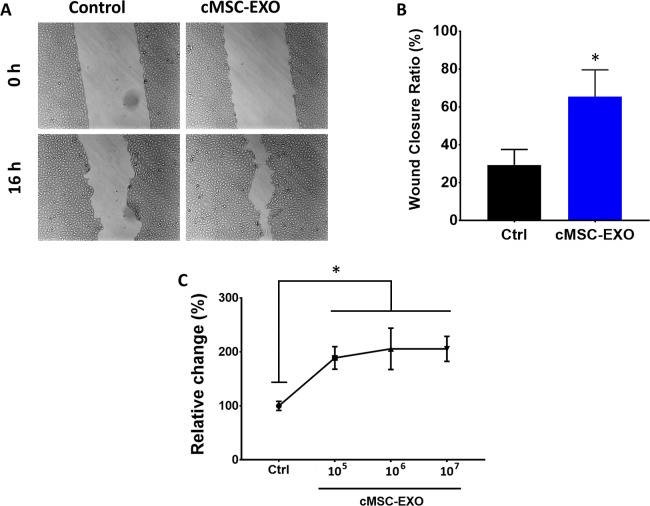
The effect of cMSC derived exosomes on epithelial wound healing and proliferation. (A, B) Scratch assay on HCECs showed significantly greater wound closure in cells incubated with cMSC derived exosomes after 16 hours. (C) MTT assay showed increased proliferation of HCECs incubated with cMSC derived exosomes after 16 hours. There appears to be a slight dose dependent effect with increasing concentration of exosomes. *Statistically significant, P < 0.05.

To assess the effect of cMSC exosomes on wound healing in vitro, a monolayer of confluent HCECs were scratched and then treated for 24 hours either with 1.0 × 10^8^ exosome/mL media or vehicle (PBS) mixed with media. Re-epithelialization in monolayers treated with exosome was significantly accelerated, with 30.1% ± 14% remaining wound area after 16 hours, compared to that of control with 72.9% ± 8% (*P* < 0.005) (Figs. 4A, 4B). Consistent with this observation, exosomes also significantly increased the proliferation of HCECs. There was a slight dose dependent effect from 10^5^ to 10^7^ exosomes/mL, but this was not statistically significant (Fig. 4C).

### Human cMSC Derived Exosomes Accelerate Murine Corneal Epithelial Wound Healing In Vivo

**Figure 5:**
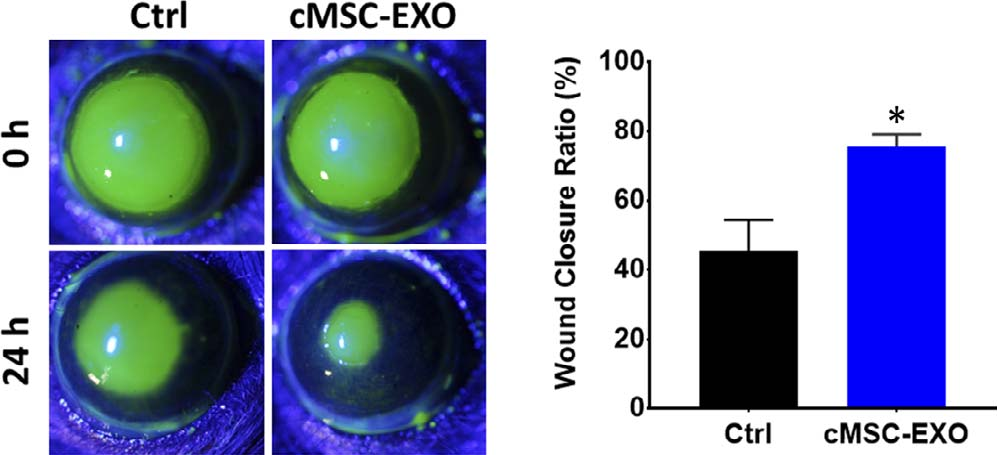
The effect of cMSC derived exosomes on corneal epithelial wound healing in vivo. Fluorescein-stained images of 2-mm wounded mice corneas, before and 24 hours after treatment with either cMSC derived exosomes or vehicle control. Exosome treated wounds had healed significantly more than control (n = 6). *Statistically significant, P < 0.05.

The effect of cMSC exosomes was evaluated in vivo using an epithelial mechanical injury model. Human cMSC derived exosomes or vehicle were applied topically to the cornea following 2 mm epithelial debridement wounds. At 24 hours, exosome treated wounds demonstrated significantly greater healing compared to control (77.5% ± 3% healed versus 41.6% ± 7%, *P* < 0.05). Representative images of fluorescein-stained wounds are shown in Figure 5.

## Discussion

Exosomes are considered a form of extracellular vesicles. They are lipid bi-layer vesicles with a size range of 40 to 100 nm that are secreted from intracellular multivesicular bodies.^20^ Exosomes contain complex molecular components, which include general and cell type specific lipids, proteins, mRNA, and miRNA, enabling them to function as vectorized, multisignaling devices.^27–29^

In the present study, we revealed the presence of protein-containing nanovesicles in our isolated samples, specifically demonstrating the expression of CD9, CD63, CD81, and HSP70, markers typically associated with exosomes.^22^ We also confirmed the absence or underrepresentation of intracellular proteins in our samples, including GM130, Calnexin, and Cytochrome-C, which should be absent or underrepresented in exosomes.^22^ The DLS results of our samples showed a bimodal distribution of particles, one in the size range defined for exosomes (40–100 nm)^20^ and another smaller curve ∼259 nm (Fig. 1). This observation is in line with previous DLS detection of exosomes.^25^ It is well known that low levels of larger particles (as low as 0.5% of all particles) can alter the intensity-weighted size distribution due to their brighter light scattering (as can be seen in Fig. 1A) and falsely magnify the amount of the large particles in DLS setting^24–26^; therefore, the results of the DLS should be confirmed and adjusted with other methods, like electron microscopy.^25,30^ Consistent with previous reports on exosomes, by electron microscopy we observed that the vast majority of these particles were within the exosome size range (under 100 nm). These observations, along with the expression of characteristic markers, strongly support the conclusion that our isolated nanovesicles were primarily exosomes.

Exosomes have been shown to play a major role in carrying out MSCs' therapeutic functions via cell-to-cell communication (“horizontal”) and modulating the molecular activities of recipient cells.^17–21,31,32^ In cutaneous wound healing, MSCs exosomes have been shown to accelerate re-epithelialization, promote proliferation, and inhibit apoptosis of skin cells in vivo and in vitro.^33^ Exosomes released from human induced pluripotent stem cell-derived MSCs have the ability of tissue repair by promoting collagen synthesis and angiogenesis.^34^ Similarly, adipose MSC-derived exosomes promote cutaneous wound healing via optimizing the properties of fibroblasts such as migration, proliferation, and collagen synthesis.^35^ Human umbilical cord MSC-derived exosomes may accelerate wound healing, in part due to the impact on collagen synthesis through Wnt-4 signaling pathway.^36^

Recently the use of exosomes in the ocular diseases have been attracting attention as well. In a recent study, Bai et al.^21^ found that human umbilical cord MSC-derived exosomes greatly reduced the intensity of ongoing experimental autoimmune uveoretinitis by reducing the infiltration of T cell subsets, and other inflammatory cells, in the eyes. Furthermore, Yu et al.^33^ showed that human umbilical cord MSC-derived exosomes ameliorate laser retinal injury via a mechanism involving MCP-1 downregulation. In another study, Han et al.^15^ showed that mouse corneal epithelial-derived exosomes fused to stromal keratocytes in vitro and induced myofibroblast transformation suggesting that exosomes may be involved in corneal wound healing.

Exosomes have several advantages over actual delivery of MSCs to the site of injury. Exosomes can be isolated readily through centrifugation techniques, providing the benefits of MSC-mediated paracrine repair without the risk of immunological rejection, malignant transformation, and obstruction of small vessels associated with cell therapy.^13^ They can be safely stored as they have excellent stable chemical properties and high biosecurity. The bi-lipid membrane of exosomes can maintain the encapsulated proteins, messenger RNA (mRNA), and microRNA (miRNA) under stable conditions to exert lasting effect.^27–29^ Therefore, they can be formulated as a topical gel or drop and can be locally administered. They can also be reprogrammed to be carriers for therapeutic agents. Finally, due to their smaller size, they are also capable of migrating deep into the corneal stroma compared to the cells.

Future studies are still needed to determine the precise mechanism by which cMSC exosomes are affecting corneal epithelial wound healing. In particular, further investigations based on their content analysis (proteins, mRNA, and miRNA) are necessary to elucidate the mechanisms of their regenerative effects.

In summary, exosomes secreted by human cMSCs can be readily isolated. They are taken up by the epithelial cells, in turn, increasing their migration and proliferation in vitro and accelerating their wound healing in vivo. Our findings suggest that human cMSC exosomes may represent a novel therapeutic approach in the management of corneal wound healing disorders.
